# Dosing intact birch pollen grains at the air-liquid interface (ALI) to the immortalized human bronchial epithelial cell line BEAS-2B

**DOI:** 10.1371/journal.pone.0259914

**Published:** 2021-11-16

**Authors:** Joana Candeias, Carsten B. Schmidt-Weber, Jeroen Buters

**Affiliations:** Center Allergy & Environment (ZAUM), Member of the German Center for Lung Research (DZL), Technical University Munich, Helmholtz Center Munich, Munich, Germany; Emory University School of Medicine, UNITED STATES

## Abstract

In real life, humans are exposed to whole pollen grains at the air epithelial barrier. We developed a system for in vitro dosing of whole pollen grains at the Air-Liquid Interface (ALI) and studied their effect on the immortalized human bronchial epithelial cell line BEAS-2B. Pollen are sticky and large particles. Dosing pollen needs resuspension of single particles rather than clusters, and subsequent transportation to the cells with little loss to the walls of the instrumentation i.e. in a straight line. To avoid high speed impacting insults to cells we chose sedimentation by gravity as a delivery step. Pollen was resuspended into single particles by pressured air. A pollen dispersion unit including PTFE coating of the walls and reduced air pressure limited impaction loss to the walls. The loss of pollen to the system was still about 40%. A linear dose effect curve resulted in 327-2834 pollen/cm^2^ (± 6.1%), the latter concentration being calculated as the amount deposited on epithelial cells on high pollen days. After whole pollen exposure, the largest differential gene expression at the transcriptomic level was late, about 7 hours after exposure. Inflammatory and response to stimulus related genes were up-regulated. We developed a whole pollen exposure air-liquid interface system (Pollen-ALI), in which cells can be gently and reliably dosed.

## Introduction

A rise in allergic diseases like asthma, hay fever and food allergies has been reported since the 1960s, which is referred to as the allergy epidemic of the 20th century [1]. Allergy research currently focuses on the identification of causative determinants to be able to reverse this increase of allergic diseases [2]. Airborne allergens like pollen are one of the main causes of allergy and may cause rhinoconjuctivitis, eosinophilic bronchitis or allergic asthma.

In real life, humans are exposed to whole pollen grains. These grains are larger than most epithelial cells (21 μm for birch, 45 μm for grass pollen) [3–5]. The aeroallergens reside inside the pollen [6] and are released within minutes after being hydrated [7], i.e. upon contact with (humid) epithelial cells. Other compounds from pollen, like PALMS derived from lipids, are on the surface of pollen and are also rapidly bioavailable [8]. Indeed, most allergic reactions occur rapidly within minutes [9] after humans are exposed to whole pollen. Immunotherapy with pollen extracts has been used for the treatment of allergic diseases since 1911 [10]. The first attempts of immunotherapy used whole pollen grains for desensitization, which resulted in significant side effects in patients [11]. Thus, from the beginning, the use of pollen extracts became the standard method for allergen-specific immunotherapy (ASIT) [10] and consequently for allergy research, either for in vivo or in vitro studies. Indeed, nasal challenges in humans use pollen extracts or standard allergen solutions [12–14]. In mice, pollen grain suspensions were used [15], but these contain extracted pollen in their solution. Newer studies with humans performed in exposure chambers use natural pollen grains [9, 16–20], and for registration of an ASIT by medical authorities studies, real-life data with exposure to whole pollen are essential.

We postulate that in working with extracts, important pharmacokinetic and pharmacological endpoints may be missed. This relates to studies on the effects of environmental pollution on human health, where the dosing of extracts to submerged cells is being challenged and slowly replaced by exposures at the air-liquid interface. For instance, exposure at the air-liquid interface (ALI) of airway epithelial cells to Diesel Exhaust Particles (DEP) and wood combustion particles was established to mimic real-life exposure to combustion derived-PM [21–23]. The combustion aerosols produced, including the reactive gases, are immediately delivered to the cells. Dosing at the air-liquid interface is technically challenging and thus costly, and not routinely carried out [22]. We propose that the same should be done for pollen exposure as pollen grains are also dosed at the air-liquid interface in real life. For Pollen-ALI (Pollen Sedimentation Chamber exposure to ALI cell culture), we know of just one system that makes whole pollen ALI exposure possible [24]. Here, variability of dosing seems to be high, probably due to the characteristics of pollen grains.

Whole pollen is sticky and thus difficult to work with as it adheres easily to any surface [25, 26]. For entomophilous pollen this is deliberate, as a sugar containing glue on pollen called “pollenkitt” enables transport of pollen on the legs of insects [27]. Anemophilous (wind pollinated) pollen, like birch pollen, do not need such a mechanism but still show rudimentary sticking characteristics [25]. In aerodynamic studies, the formation of clusters of pollen upon resuspension (which sediment much more rapidly than single pollen) has been reported [28]. Overcoming both loss to the instrumentation and clustering are among the main obstacles that were not yet solved.

Our goal was to establish a whole pollen sedimentation chamber, and expose immortalized human bronchial epithelial cell line BEAS-2B to real-life doses of intact pollen. We developed methods that allowed resuspension of pollen as single particles, developed an assay to study the adhesion of a pollen grain to different surfaces (to control for pollen loss), and created a chamber that allows a reproducible and even distribution of the whole pollen.

We exposed BEAS-2B cells to real-life pollen doses at ALI, at doses that are low compared to the often used pollen extracts. Transcriptional analysis of BEAS-2B exposed to pollen for different lengths of time showed the largest response at about 7 hours after pollen exposure, with pathways related to inflammation and immune responses being up-regulated. This study demonstrates the development of a Pollen-ALI that enables a better understanding of factors that control the etiology of allergic diseases.

## Materials and methods

### Development of the Pollen Sedimentation Chamber

Birch pollen were freshly collected in Munich, in 2018, sieved through 100 and 70 μm Mesh (CISA Cedaceria Industrial, Spain) and stored as aliquots at -70°C. Aliquots of the pollen were allowed to reach room temperature for 2 hours before opening a vial. Relative humidity and temperature were recorded before weighing of pollen. The amount of allergen released from pollen was determined by ELISA and was 3.7 pg/pollen grain, in agreement with the literature [29].

**Fig 1:**
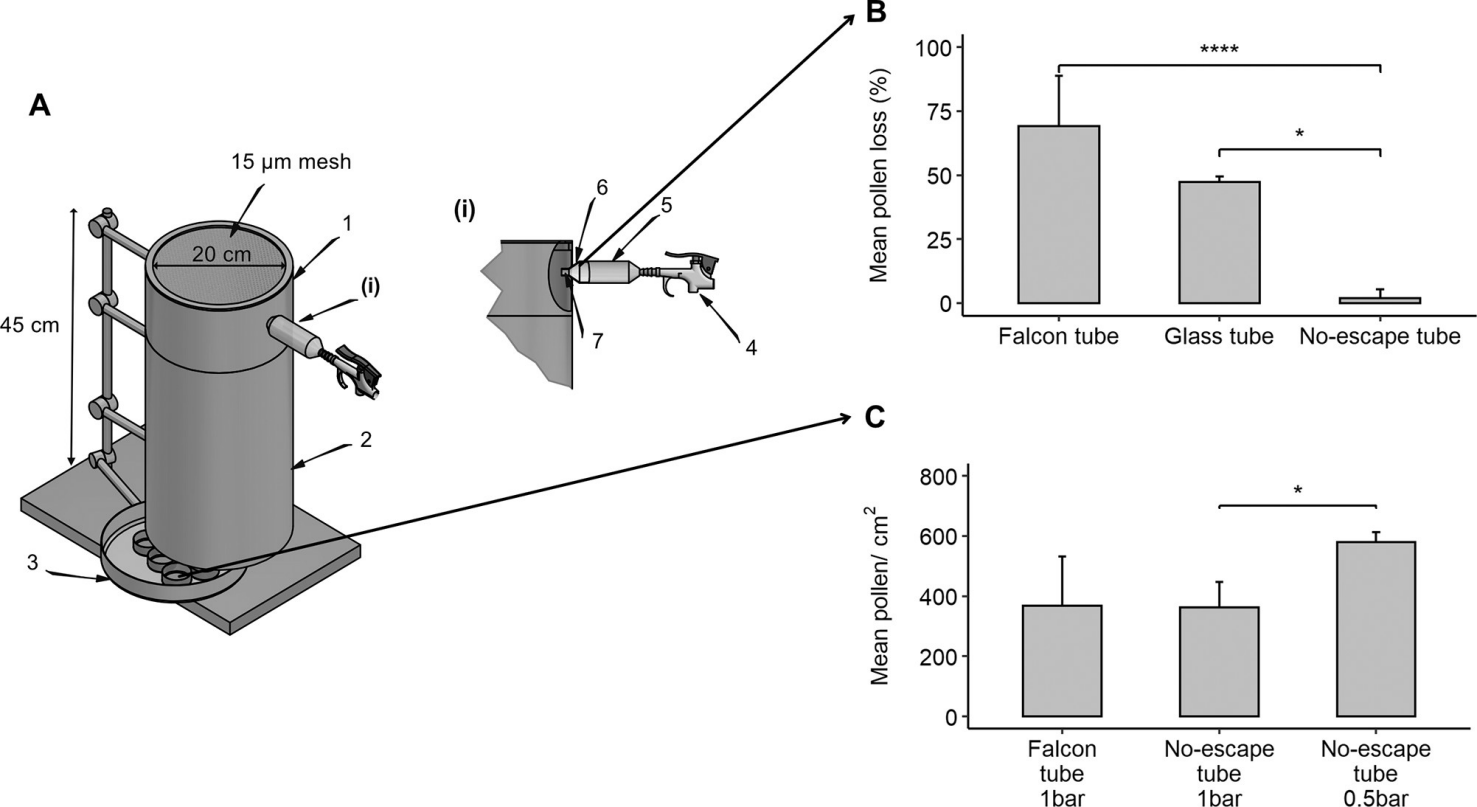
Characteristics of the air-liquid pollen sedimentation chamber. (A) dispersion (1), sedimentation (2), and cell loading chamber (3). i) Detail with a pressured air gun (4), pressure shock absorber (5), “No-escape” loading tube (6) and location of the “to be dosed” pollen (7). (B) Effect of different loading tubes on the pollen loss before entering the chamber. (C) Effect of dispersion pressure on the pollen deposition. N = 4 in all cases, *p<0.05, *** p<0.001.

Aluminium tubing with a diameter of 20 cm was used for chamber construction. Birch pollen are about 21 μm in diameter and so a stainless-steel mesh of 15 μm (Utah Biodiesel supply, USA) was used to allow exchange of air but not pollen (Fig 1A).

Pollen deposition on undesired places was checked by using Vaseline (Bombastus-Werke, Freital, Germany) coated Melinex® tape (Burkard Manufacturing Co Ltd, England) followed by microscope analysis and manual counting.

Pollen deposition on cells was performed for 1, 2, 4 and 10 mg whole birch pollen and quantitated with circular Vaseline coated glass cover slides, with the same diameter as a 6-well insert (24 mm), distributed on the same place where each 6-well insert would have been. In addition, two 12 mm diameter cover glasses were placed inside each culture plate to be used for dose assessment, also when exposing cells. Pollen on cover glasses were counted with a light microscope (Leica Microsystems GmbH, Germany) using 100x magnification. Microscope pictures of pollen were performed with EVOS® FL Auto (Life Technologies) and treated with ImageJ Software (National Institute of Health, USA). Two lines crossing the cover glass were counted, representing an area of 0.48 cm^2^. Different counting methods were tested and the two lines method resulted in a representative estimate of the distribution of the pollen on the cover glass. Pollen loss was calculated by relating weighted pollen to recovered pollen on a cover slide of 0.48 cm^2^ in relation to the surface of 283.5 cm^2^ of the bottom of the chamber, using a pollen weight of 7 ng/grain pollen [3, 4, 26].

Lack of adhesion of pollen to surfaces (protective coating) was tested with the “Slide-friction-test”: each surface was declined 45° and about 2 mg of pollen were applied with a brush to a marked position. The distance that pollen had moved down the slope from this mark was measured. The longer the distance, the less the pollen adhered to this surface or coating (S1A Fig).

### Air-liquid-interface exposure to whole birch pollen

**Fig 2:**
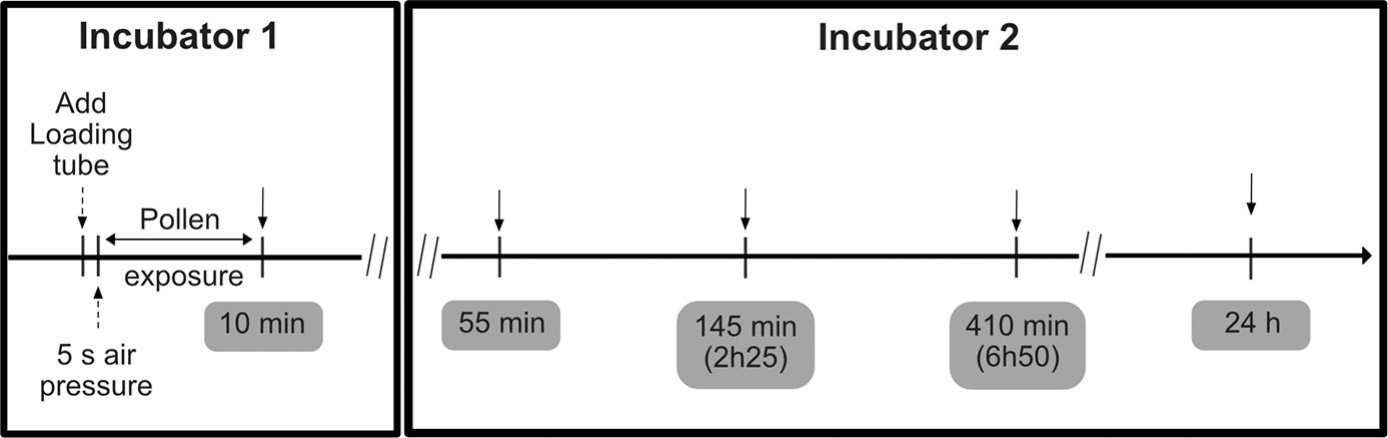
Experimental design of dosing pollen on BEAS-2B cells at ALI. Incubator 1 contained the complete Pollen-ALI where the pollen was dosed to the cells. Pollen sedimentation was completed after 10 min and the exposed cells were transferred into a second incubator and kept at ALI conditions for the indicated times. Both incubators were at 37°C and 5% CO_2_.

The immortalized human bronchial epithelium cell line BEAS-2B (ATCC® CRL-9609™) was cultured as in [22]. For pollen exposure, 0.4x10^6^ cells were seeded on transferable 24 mm Transwells® inserts with a 0.4 μm pore polyester membrane (Corning, Tewksbury, USA) and kept in submerged conditions for 24 hours. At all times, cells were cultured on pre-coated flasks and inserts using a solution with 0.01 mg/mL fibronectin, 0.03 mg/mL bovine collagen type I and 0.01 mg/mL bovine serum albumin - all from Sigma-Aldrich^®^ - Germany, according ATCC’s recommendations. Basal medium was then changed and the apical medium was removed for ALI conditions. Cells were then kept in air-liquid interface conditions for 24 hours before pollen exposure. Confluence of cells was checked by light microscope before pollen exposure and only inserts with more than 85% confluence were used. Cells were inserted into the Pollen-ALI, in Incubator 1 that was kept at 37°C and 5% CO_2_. BEAS-2B cells were exposed to 10 mg of whole birch pollen (or mock exposed) by dispersing the pollen with 0.5 bar pressured air into the chamber (Jun-Air model-6 Compressor, Redditch, UK). Sedimentation was allowed to proceed for 10 min. Exposed cells were then returned to the original incubator and further incubated under ALI conditions for the indicated times (Fig 2). The same conditions were used for the mock exposure (devoid of pollen).

Cell viability and toxicity were assessed by the Alamar Blue® (Thermo Scientific™, UK) and LDH-Cytotoxicity Detection Kit assay (Roche Diagnostics GmbH, Mannheim, Germany), respectively, according to the manufacturer’s instructions.

Cells always kept in “Incubator 2” (not exposure or mock exposure) were used as a negative control and results for the LDH and Alamar Blue assay were normalized to these cells. Cells cultured in the same conditions were lysed with 2% Triton-X 100 (Roth, Karlsruhe, Germany) at the end of each exposure period and used as positive control. Absorbance was measured with a microplate reader (Epoch, Biotek Instruments, Inc., USA) for both toxicity and viability assays (490 nm and 600 nm, respectively).

### Whole-genome expression analysis

Transcriptomic analysis of the BEAS-2B cells were performed for all incubation times. Immediately after each exposure time, cells were lysed using Buffer RLT of the RNeasy Plus Mini Kit (QIAGEN, Hilden, Germany), with additional 1% ß-Mercaptoethanol (Roth, Karlsruhe, Germany). Total RNA was extracted according to the manufacturer’s protocol. Quantity and quality of the RNA was assessed with an UV-vis spectrophotometer (NanoPhotometer® N60, IMPLEN, Munich, Germany) and the Agilent 2100 Bioanalyzer RNA Nano chip (Agilent Technologies, Waldbronn, Germany), respectively. RNA samples with RIN higher than 8 were spiked (One-Color RNA Spike-in Kit, Agilent Technologies, Waldbronn, Germany), Cy3-labelled (Low Input Quick Amp Labeling Kit, one-color, Agilent Technologies, Waldbronn, Germany) and purified on RNeasy mini spin columns (QIAGEN). Generated Cy3-labelled cRNA was hybridized on One-Color SurePrint G3 8x60K Human gene expression arrays (Agilent Technologies, Inc, Waldbronn, Germany), according to the manufacturer’s protocol. Microarray slides were scanned (Agilent Microarray Scanner, Agilent Technologies, Waldbronn, Germany) and data were extracted using Feature Extraction Software Version 11.0.1.1 (Agilent Technologies). Transcriptomic data was analysed using the statistical programming environment R (version 4.0.2) [30]. In detail, pre-processing of the microarrays was performed using the Limma package [31]. Differential Expressed Genes (DEG) were compared with identically but without pollen treated cells as negative controls (i.e. the pollen were prevented from entering the wells with a 15 μm Mesh), using the eBayes method. Cut-offs were p<0.05 (adjusted p-value with Benjamini-Hochberg) and ≧ 1.1-fold up- or down-regulation. Microarray data discussed here were submitted to the National Center for Biotechnology Information (NCBI) Gene Expression Omnibus (GEO) and can be accessed through GEO Series accession number GSE179942.

### Validation of microarray results by qRT-PCR

Selected genes from different enriched GO terms related to allergy, inflammation response (CXCL2, IL1B, IL24, IL6, TNFAIP3, SOCS3) and cell junction (ICAM1), which were differentially expressed by microarray analysis, at different time points, were validated by quantitative real-time PCR. Reverse transcription of total RNA samples used for microarray analysis was performed with the High Capacity cDNA kit (Applied Biosystems, USA), according to manufacturer’s instructions. qRT-PCR was performed with ViiA 7 Real-Time PCR System (Applied Biosystems, USA), using the FastStart Universal SYBR Green Mastermix (Roche, SUI). QIAGEN human primer Assays (QIAGEN, Hilden, Germany) were used, including endogenous controls (ß-actin and 18S). The mRNA expression of the selected genes was normalized to the endogenous controls and relative quantification was calculated using the comparative Ct method (2^-ΔΔCT^), as described in [32]. All amplifications were carried in duplicate and fold changes in mRNA expression of pollen-exposed BEAS-2B cells were compared with control cells (not exposed to pollen).

### Statistical methods

Independent sample T-test was performed for data with two groups. ANOVA was used for multiple groups, like pollen loss and dose, with the adequate Post-Hoc tests for each hypothesis. Benjamin-Hochberg p-value correction was used for all tests. Unless stated, Standard Deviation (SD) was used to report the variability of the mean.

All tests and cell exposure experiments were reported from at least 3 independent repetitions on different days. For the transcriptome analysis, the enrichment analysis was performed using the Metascape web-based portal [33]. Enriched terms were selected based on the GO (Gene Ontology) Biological Processes and pathways from different databases (Canonical, Hallmark, Reactome, KEGG, WikiPathways and BioCarta), with the cut-off of minimum 3 genes, fold change > 1.2 and p-value < 0.05.

## Results

### Establishing a Pollen Sedimentation Chamber

**Fig 3:**
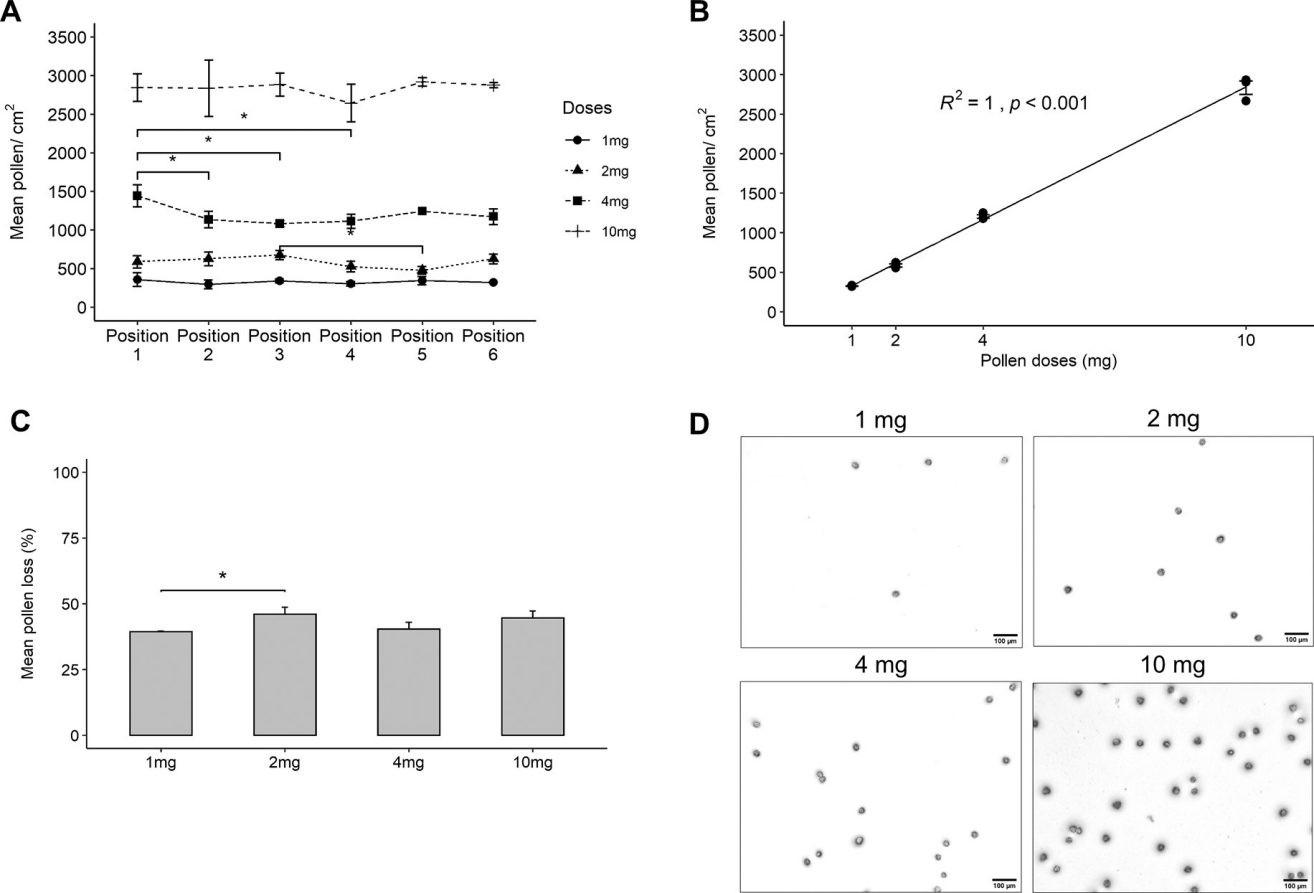
Distribution and dose effect curve of pollen dosing. (A) pollen dose depending on dose and position in the chamber, (B) dose effect curve, (C) loss of pollen to the chamber depending on dose, (D) Microscope pictures of surface distribution of pollen, for the different doses, within single positions. N≥ 3 in all cases.

The presence of pollen clusters during resuspension, one of the main problems we wanted to avoid in the pollen chamber [28], was rarely observed (Fig 3D) and turned out to be a minor issue.

We developed techniques to minimize pollen loss to the surfaces of the instrument. The chamber was made of 5 mm thick aluminium to reduce static electricity. However, when loading 2 mg of birch pollen into the chamber, more than 53 ± 18% of the pollen was lost (S1B Fig). Adhesion of pollen on the surfaces of the chamber will lead to reducing the amount of deposited pollen on the cells. We established the “Slide-friction-test“ to check lack of adhesion of pollen to any surface in order to find an optimal coating material (S1A Fig). With this technique, we tested many surfaces and coatings and concluded that coating with Teflon® (PTFE) resulted in the least adhesion of pollen. Still, pollen did adhere to the surface. To further minimize this adherence we used different resuspension air pressures, and used Vaseline coated strips (for maximum adhesion) to check the degree of contact to the walls (see below).

The Pollen Sedimentation Chamber was then coated with PTFE, which reduced the pollen loss and increased the deposited dose by 13% (p = 0.58, S1B and S1C Fig). Other effects than surface composition were also important for pollen loss and exposure variability. We therefore investigated the effect of the design - how pollen entered the chamber - and different loading methods were tested.

Three different loading tubes (out of many) are discussed. A 50 mL Falcon® tube with one hole at the bottom and no lid was used. Pollen was weighed in aluminium foil that was then placed inside the Falcon® tube. A wide mesh was created in the loading port of the Pollen Sedimentation Chamber to allow pollen to enter but not the aluminium foil. Before use, the Falcon® tube was cleaned with acetone to remove possible dirt and other debris. As the polypropylene of the Falcon® tube showed electrostatic or other properties that made the pollen adhere to the surface, the same construction out of hexamethyldisilanized glass was made, to increase hydrophobicity and reduce possible loss of pollen to the walls of the tube. None of the adjustments substantially reduced pollen loss. Resuspension of pollen by generating a cloud of pollen, before entering the sedimentation chamber, resulted in a marked loss to the walls of all loading tube designs. We then opted for creating the pollen cloud inside the pollen chamber itself. To do so, we created the “No-escape tube”. This was a 5 mm by 50 mm long polystyrene tube. Pollen was weighed into the tube, which was then placed at the entrance of the chamber (Fig 1A). Pressured air (into a Falcon® tube to absorb any air pressure shock) resuspended the pollen (Fig 1A).

Fig 1B shows the pollen loss in the loading tube for the three loading tubes described here. The loss of pollen to the loading tube is almost reduced to zero when using the “No-escape tube”. The glass tube retained 47.4 ± 2.1% of the pollen and the Falcon® tube 69.1 ± 19.7%. When using the glass tube, clusters were seen on the bottom of the chamber, which did not happen with the other loading tubes. With the “No-escape tube” we could reduce the loss to the dosing tube, i.e. the loss before pollen entered the chamber. However, inside the chamber, a substantial loss remained.

We then determined inside the chamber whether the air pressure used could influence pollen adherence to the opposite site of loading. Dual sticky Melinex® tapes coated with Vaseline were distributed inside the chamber as in S2A Fig. Air pressure used to propel pollen into the chamber turned out to be a critical step. Two different air pressures are shown: 1 and 0.5 bar (S2 Fig).

S2B Fig shows the same area of the tape that is on the opposite site of the loading port (T3), for both exposures. At the higher air pressure (1 bar) more pollen was detected on the opposite site of the loading port compared to 0.5 bar. Low air pressure decreased pollen loss significantly by around 18 ± 6% (n = 4, p = 0.047). Consequently, lower pressure led to a significant increase of deposited pollen to about 62.7 ± 23.1% pollen/cm^2^ on the inserts (Fig 1C, no tape inside the chambers). In addition, the variability between exposures was also reduced. Thus, at the higher air pressure, pollen can be propelled against the opposite wall, where they stick with variable loss. More gentle dispersion (like the wind does with catkins), results in less loss and a more reproducible dispersion. Under these conditions, pollen does not seem to stick to each other but stick very well to all other surfaces. Our efforts resulted in a significant, but reproducible loss, enabling dosing cells at a target pollen concentration.

### Determination of pollen doses

We then determined if dosing was even over the cell loading surface. Each well of a 6-well culture plate was replaced with cover glasses, covered with Vaseline, with the same diameter as an insert (24 mm). Although Vaseline might be different from a cell surface, fixing and counting pollen on the cells was impossible due to a “Tsunami effect”: pollen were partly washed away during the fixing procedure. No major statistically significant differences were seen between the six positions (Fig 3A). Higher doses of pollen gave the same variability on the deposition of pollen.

A dose-effect curve (a “dose in” versus “dose deposited”-curve) was established (Fig 3B). The curve was linear with dose (r = 0.99, p>0.001) with a substantial but reproducible and low variability loss of 42.6 ± 3.5% (Fig 3C). The density of the obtained pollen distribution is visualized in Fig 3D.

### Manipulation stress of BEAS-2B in the Pollen Sedimentation Chamber

**Fig 4:**
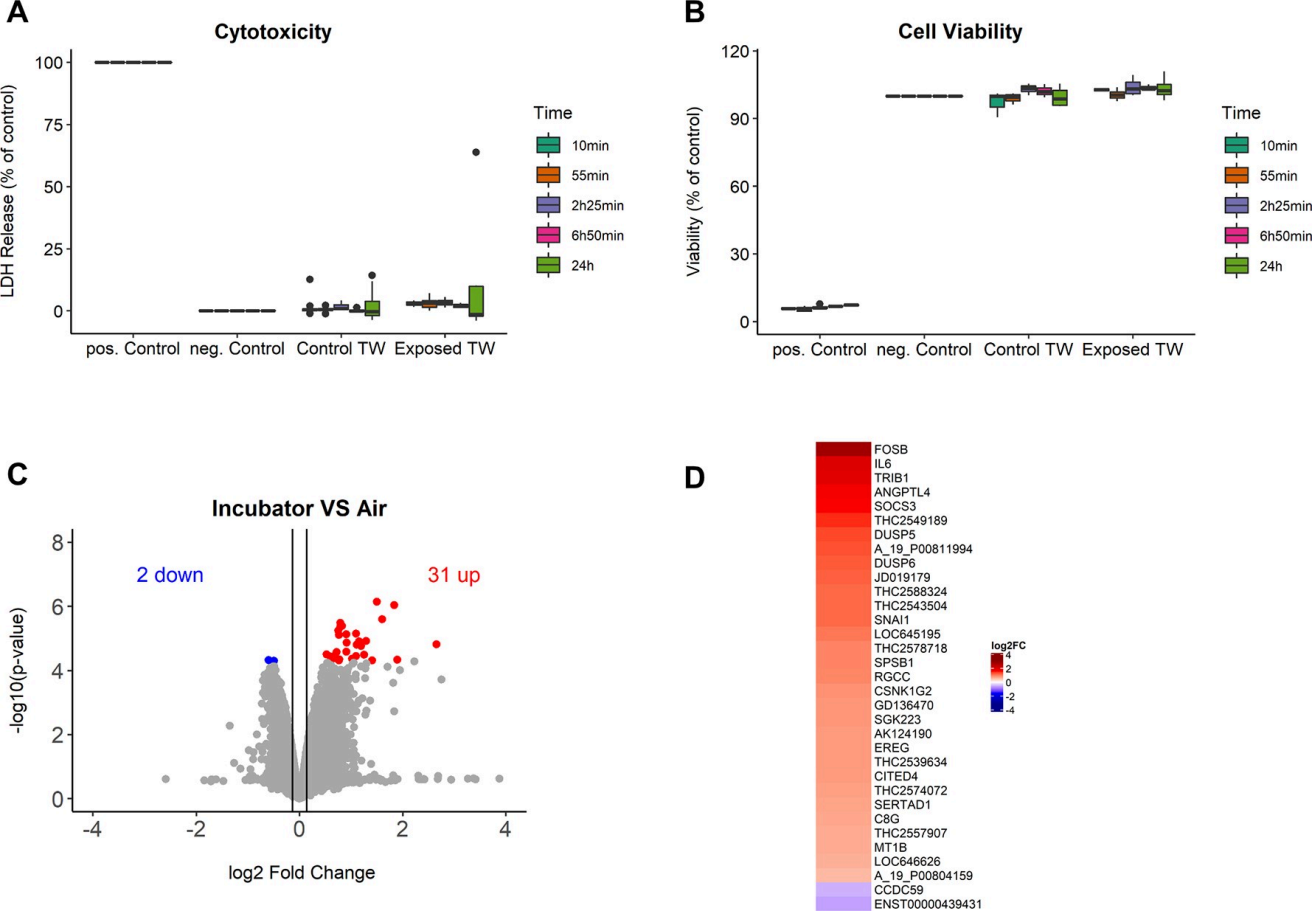
Effect of whole pollen and mock exposure in cellular health of BEAS-2B. (A) cytotoxicity (LDH), after dosing 10 mg pollen to BEAS-2B cells, and (B) cell viability of the cells (Alamar blue), for the different incubation times. (C) volcano plot and (D) heatmap of the effect of mock experiments (no pollen) on BEAS-2B, analysed by genome-wide gene expression arrays after 2h25 min. Incubator cells are used as control. Significant regulated genes were marked in color. N≥ 3 in all cases.

The reaction of cells to the procedure (manipulation stress) was tested by mock exposing cells (according to Fig 2) without using pollen (pressured air was applied to the loading tube) versus the same cells that never left the incubator. A transcriptomic analysis was performed for the 2h25 min incubation time, between incubator 2 as control and the mock exposed cells. Indeed, manipulating the cells showed some reactions (Fig 4). The cells, however, showed no viability loss or any toxicity upon mock exposure at point of time (Fig 4A and 4B). A limited number of genes (31) were up-, and 2 genes down-regulated after 2h25 min post-exposure. The genes up-regulated are related to biological processes, as of protein phosphorylation (*DUSP6*, *DUSP5*, *SOCS3*, *IL6*, *RGCC*, *SERTAD1*, *EREG*, *TRIB1*) and response to stimulus (*DUSP6*, *DUSP5*, *MT1B*, *SOCS3*, *IL6*, *CSNK1G2*, *FOSB*, *CITED4*, *SNAI1*, *RGCC*, *C8G*, *ANGPTL4*, *EREG*, *TRIB1*). Fig 4D shows a fold-change heatmap of the genes in detail. The up-regulation of these genes was considered a sign of (minimal) stress for the cell cultures, inherent to any changes from their normal culture conditions. Nevertheless, in all subsequent experiments, mock-exposed cells were included and the mock effects were subtracted.

### Exposure of BEAS-2B to whole birch pollen

The Pollen-ALI function was tested by exposure of the immortalized human bronchial epithelial cell line BEAS-2B, to whole pollen with a dose (pollen/cm^2^) similar to realistically to be expected in a human nose. The reaction of the immortalized human bronchial epithelial BEAS-2B cells might not be the same as the reaction of human nasal epithelium. When loading 1 mg of birch pollen into our chamber we can reproduce a normal peak birch pollen dose (historical data 2004-2018 pollen monitoring station Biederstein, Munich, Germany). With 4-10 mg, we can mimic a higher but in nature occurring higher pollen dose, as can be encountered in the heartland of birch trees in Europe i.e. Russia (see discussion). All exposures included a concomitant mock control exposure (i.e. 15 μm mesh protected cells). The pollen we used liberated around 3.7 pg Bet v 1 per pollen grain [29].

**Fig 5:**
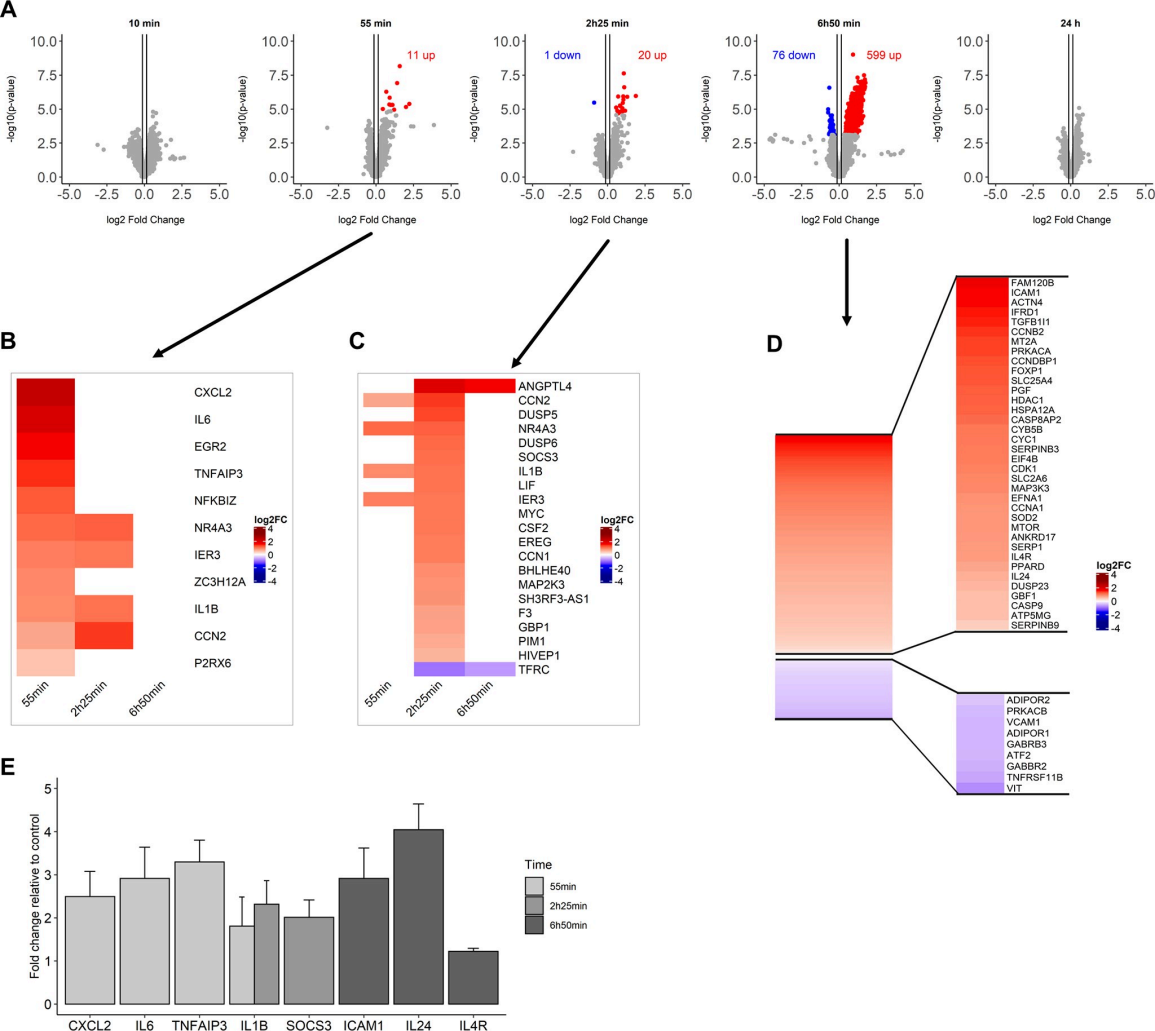
Transcriptomic analysis of the effect of 10 mg whole birch pollen on immortalized human bronchial epithelial BEAS-2B cells, at the air-liquid interface. (A) volcano plots and (B, C) heatmaps of all significant regulated genes for the 55 min and 2h 25 min incubation times, respectively (D) heatmap of all the significant regulated genes for 6h 50 min incubation time, with focus on the most relevant genes. N≥ 3 in all cases. (E) mRNA expression of selected genes by qRT-PCR that were expressed in transcriptome data. N ≥ 2 for all experiments.

In Fig 5, the differential gene expression from microarray data is displayed for the cells exposed to 10 mg whole birch pollen. At early time points, a limited reaction was observed: nothing at 10 min, 11 up-regulated genes at 55 min, 20 up- and 1 down-regulated at 2h 25 min (all with mock genes quantitatively subtracted). A major reaction was detected after about 7 hours when about 600 genes were up- and 76 down-regulated. After 24 hours, transcriptomic changes in BEAS-2B cells had reduced to zero.

At 55 min we observe an up-regulation of genes related to inflammatory response (*TNFAIP3*, *IL6*, *IL1B*, *CXCL2*, *ZC3H12A*, *IER3*, *EGR2* and *NFKBIZ*).

In general, the genes up-regulated at 2h 25 min are in concurrence with the ones expressed at 55 min. Genes related to cytokine signalling, inflammatory response and MAPK-pathway related (*CCN2*, *CSF2*, *DUSP6*, *DUSP5*, *EREG*, *F3*, *GBP1*, *IL1B*, *LIF*, *MYC*, *PIM1* - known as *LONP1* -, *MAP2K3*, *SOCS3*) were up-regulated when cells were exposed to whole birch pollen.

After 6h50 min incubation time, the highest number of up-regulated genes were obtained. A Metascape analysis was performed to better understand the whole gene expression. The top Gene Ontology (GO) enriched terms are shown in S3 Fig. GO terms related to signalling, response to stimulus, adhesion and immune system process are differentially regulated. Focusing on these groups of GO terms and according to the pathways related to them, a set of genes were examined in more detail (Fig 5D).

Genes related to cell junction were differentially regulated, with most of the genes being up-regulated, like *CDK1*, *CEP* family genes, *ICAM1* and *ACTN4*. Pathways related to intracellular signalling pathway, stress response, *VEGF* and *MTOR* related, the latter showed in the last years to be related with allergy [34, 35]; and oxidative phosphorylation are up-regulated, like *FOXP1*, *SERPIN* family, *SOD2*, caspase family, *PGF*, *CYB5B*, *SERP1* and *SLC25A4*. Genes related to immune response, NOTCH signalling and TNF signalling via NFKB were mostly still up-regulated after 7 hours exposure to whole pollen, with the expression of genes like *IL24*, *IL4R*, *TGFB1I1*, *MAP3K3*, *MTOR*, *PPARD* and *SLC2A6*.

A representative selection of genes, related to different pathways and regulated at different time points, were validated by qRT-PCR (Fig 5E). *CXCL2*, *IL6* and *TNFAIP3* mRNA expression was higher at BEAS-2B cells exposed to pollen, compared to control cells, at 55 min. *IL1B* showed a higher expression at 2h 25 min, as observed in the transcriptomic data. Also at 2h 25 min, the expression of *SOCS3* was of 2.0 fold (± 0.4) at exposed cells, compared to control cells. For the 6h50 min time point, three genes were validated with qRT-PCR: *ICAM1*, *IL24*, *IL4R*, of which *IL24* shows the highest fold change among all (4.05 ± 0.6).

## Discussion

In the present study we show that even a high (but still natural) pollen exposure results in sparse transcriptomic changes in a bronchial epithelial cell line (Fig 5), with major changes happening 7 hours after exposure. The fast reaction that normally occurs in humans is due to mast cell activation. However, the degranulation of mast cells occurs when an individual is already allergic sensitized. Before sensitization, as in our case, mast cells do not react and the epithelium is the first location where the reaction of an individual to pollen occurs.

In nature, by evolutionary construction of the catkins that are responsible for pollen dispersal, pollen fly as single particles despite their stickiness. The main problem that we anticipated while developing the Pollen Sedimentation Chamber was that pollen would stick to each other forming clusters upon resuspension, as reported by [28]. Surprisingly, in contrast to [28], with our system we seldom encountered pollen clusters in any of the samples analysed, which we think is due to pressured air being used for resuspension (Fig 3D). The second consequence of pollen stickiness is their loss to any surface of the instrument. Indeed, this was a major obstacle we had to overcome. Another problem was the expected rupture of birch pollen by pressured air, as reported by [36]. However, we did not observe this using our experimental design.

A cylinder form was chosen to reduce the number of collisions of the pollen to the walls. The walls were made of aluminium to reduce static electricity, which could make pollen adhere easily to the surface [25, 37]. Applying high positive or negative charge (+/- 1000V) to the chamber walls versus a plate underneath the cells did not result in a change in pollen loss. Our main assumption was that contact of pollen with walls had to be avoided. Due to the inertness of a large particle (like pollen), any disturbance in airflow pattern would increase impaction and loss of the particle to the walls, especially when we consider the boundary layer effects of such adherent particles as pollen [25]. We thus opted for horizontal dispersion of single particles using pressured air, with a vertical straight-line sedimentation onto human cells. Indeed, creating a “puff” with pressured air resulted in single pollen (no clusters). The “puff” itself was directed away and 45 cm from the cells. Above the “puff” generator, a 15 μm mesh allowed pressure exchange, but stopped birch pollen from escaping. We believe that no air of the puff reached the cells. The falling speed of birch pollen is about 1.5 cm/s [4] and sedimentation upon cells then resulted in an even but gentle distribution. Although we could not prove that the contact of pollen with cells was gentle, we think that compared to human inhalation were impacting can be substantial (nose breathing 750 cm/s [38]), an impacting speed of 1.5 cm/sec in our system compares favourably. In the human nose, deposition is horizontal with impaction but with the presence of mucus, not produced by the cell line used in the study. Thus, due to mucus, impacting directly on cells is limited. We chose to avoid impacting insults on cells and dosed gently by gravimetric sedimentation. Indeed, cells dosed according to this set-up did not react at all at the transcriptome level in the first 10 min, and limited later on.

Optimization of our Pollen Sedimentation Chamber showed that several, sometimes small, improvements together were needed to get an acceptable test system. Low resuspension air pressure, coating of the surface, and geometry of how and where pollen enters the chamber were critical steps. With our design, an even distribution of deposited pollen was obtained, with a linear dose effect curve (Fig 3). Still, the loss of pollen to the chamber was about 40%. The same amount of loss was seen in [36], for a wind-induced birch pollen rupture system. Because the loss of pollen was reproducible and linear, we could obtain any pre-set dose of pollen on cells.

Our proposed design resulted in little effect on cells when mock exposed, i.e. the manipulations needed to perform a Pollen–ALI exposure resulted in an up-regulation of only 31 genes using genome wide transcriptome screening. The highest up-regulated genes were *FOSB*, *IL6*, *ANGPTL4* and *SOCS3*, all related to cellular stress. Compared to pollen exposure (see below) the reactions were limited and unspecific. We conclude that the 10 min manipulation and transport of cells was of limited influence on the toxicity, viability and transcriptome of the cells. Nevertheless, a mock control in the same experimental run was always used to compare exposed cells. Mock exposure was obtained by protecting the cells with a 15 μm mesh prevented pollen from entering, but had no effect on the “air pressure puff” we generated as this puff would never reach the cells.

In normal life, humans breathe about 14 m^3^/day [39]. Birch pollen in Munich (Station Biederstein), between 2003-2018, showed a peak concentration of 3,500 pollen/m^3^ (24h average). Then, 14m^3^ inhaled air with 3,500 pollen/m^3^ onto the nasal cavity of 160 cm^2^ [40], results in a dose of 306 pollen/cm^2^. In Russia, where most birch trees grow [41], peaks of 10,000 – 20,000 birch pollen/m^3^ are common [42], resulting in a possible dose of about 1,000-2,100 pollen/cm^2^. We dosed 3,000 pollen/cm^2^. Thus, our employed dose is high, but not unrealistic and still resulted in few cells being exposed to pollen (Fig 3D). It also compares favourably to submerged exposure, where doses often up to 10 mg/ml pollen and higher are being used [43, 44]. If our cells would be in submerged conditions (assuming 100 μl/cm^2^), we would dose around 0.2 mg/ml.

We used the bronchial epithelial derived cell line BEAS-2B as a model for reaction of the airway epithelium to pollen [45]. Although bronchial and nasal epithelium are not the same, the concept of an united airway seems to be valid and shows a correlating reaction between both tissues [46]. Of course, results detected in vitro need to be confirmed in vivo, for which we are developing the methodology (see https://eithealth.eu/project/adapt/). The cell line shows, besides the absence of cilium and mucus, limited tight junctions [47] and is frequently used to test allergic inflammation [48]. When dosing whole pollen at the ALI, and comparing with control cells, transcriptomic changes were limited. Pollen release allergens (and mediators) within minutes of hydration, but little effect was observed (see Fig 5). The highest expression of genes was seen at around 7 hours. Although other techniques like proteomics and lipidomics might pick up faster reactions, earlier times at the transcriptome level show an acute-phase response of the BEAS-2B after whole pollen exposure.

The main enriched GO terms involved in allergy observed in our study, were related to inflammation response, cellular stress and cell junction. *IL1B* is an important mediator of the inflammatory response, involved in T-cell and cytokine activation by inducing mediators like *TNF* and *IL6* [49–51], the latter also up-regulated in our exposure and induced after allergen challenge in another study [52]. TNFAIP3 and CXCL2, also related to inflammatory responses, were up-regulated at 55 min after pollen exposure and are known to be regulated in allergic diseases [53–57]. At 6h50 min, the increased expression of genes related to cell junction was observed, like *ICAM1* which is known to be induced in BEAS-2B exposed to mite and pollen allergen, playing a role in epithelial transmigration of neutrophils by a specific anchoring [58–62]. *IL24*, up-regulated 6h 50 min after pollen exposure, is associated with inflammation [63]. *IL4R* was also up-regulated in our pollen exposed cells, thus the cell can respond to *IL4* and *IL13*, inducing type 2 inflammation [64]. SOCS3 is part of the SOCS family, which are important modulators of inflammation and cytokine signalling, and were expressed in nasal epithelium of patients with allergic rhinitis [65]. BEAS-2B exposed to pollen after about 7 hours, seem to react towards the restoration of normal epithelium. After 24 hours, cells exposed to whole birch pollen had restored and showed no transcriptomic changes compared to control cells anymore.

The Pollen-ALI showed a reproducible and uniform pollen dosing to cells, despite a 40% pollen loss. The pollen dose we used was high but still a real-life pollen peak dose. Our high dose is still very low compared to other non-ALI studies. Such low doses of pollen have not previously been used in vitro or in animal exposure studies. When looking at the microscopic pictures of deposition of the whole birch pollen, only a few cells actually had a direct contact to a pollen grain, illustrating that few pollen can induce a pro-allergic reaction in real life exposure.

We did not find a significantly different regulation of *IL-33*, *IL-25* or *TSLP* genes that are accepted to be important in allergic regulation. This could have many reasons, but also emphasis the point that using a low dose of whole pollen at ALI could induce different reactions than expected. The use of epithelial or other cells in the search for early factors that could predispose individuals to becoming allergic are a promising target, as these cells form the first contact between environment and the immune system.

Our experiments show that the reaction of the epithelium was limited, but seems to induce a “danger-signal”. Danger signals are augmenting immune responses and are often needed when deliberately making animals allergic or when desensitizing humans. We expect that danger signals in humans will do the same as in animals.

## Conclusions

We designed and evaluated a system to dose whole pollen to cells at ALI. Although 40% of pollen was lost to the instrument, we were able to obtain a reproducible, equally distributed and gentle pollen dosing on immortalized human bronchial epithelial BEAS-2B cells.

The cells exhibited limited reaction to mock exposures. However, when dosing whole pollen, transcriptomic changes did occur. These reactions were few at 10 and 55 min after dosing. Differential regulation peaked at about 7 hours after exposure. The pathways involved were in the beginning mainly inflammation and evolved later into general cell activation, i.e. whole pollen induced a danger signal which have an adjuvant effect on allergic inflammation. After 24 hours reactions had ceased.

We imagine to use this system of low dose pollen at ALI for investigating the synergistic effects of environmental factors on pollen exposure in making individuals allergic.

## Supporting information

S1 Fig Effect of surface coating on pollen loss. (A) test system “(“slide-friction-test”) to pre-test the effect of a surface change on pollen stickiness (B) effect of a PTFE coating on pollen loss to the surfaces of the Pollen Sedimentation Chamber and (C) effect of coating in pollen deposition on the bottom of the Pollen Sedimentation Chamber. N≥ 3 in all cases.
(PDF)

S2 Fig Effect of air pressure on pollen loss. (A) detail of dispersion chamber with route of injected pollen when loading through entrance (1) and examples of Melinex(R) glue tapes (2), (B) sedimented pollen in the position T3, on the dispersion chamber, depending on air pressure. A higher pressure resulted in pollen being propelled against the walls and mesh, where they stuck and were lost.
(PDF)

S3 Fig Top enriched GO Terms at 6h50 min of BEAS-2B exposed to whole birch pollen. Metascape analysis of the significant regulated genes was performed. GO Terms related to Biological Processes are illustrated.
(PDF)

S1 Appendix Values used to build graphs. (DOCX)

S2 Appendix Metascape enrichment Top-GO cluster analysis for the up-regulated genes, at 6h50min. (CSV)

S3 Appendix Metascape enrichment Top-GO cluster analysis for the down-regulated genes, at 6h50min. (CSV)
