# Supplementary figures and images for: Dosing intact birch pollen grains at the air-liquid interface (ALI) to the immortalized human bronchial epithelial cell line BEAS-2B
Source: PLoS One. 2021 Nov 16;16(11):e0259914. doi: 10.1371/journal.pone.0259914 (PMC8594808; doi:10.1371/journal.pone.0259914)

**A**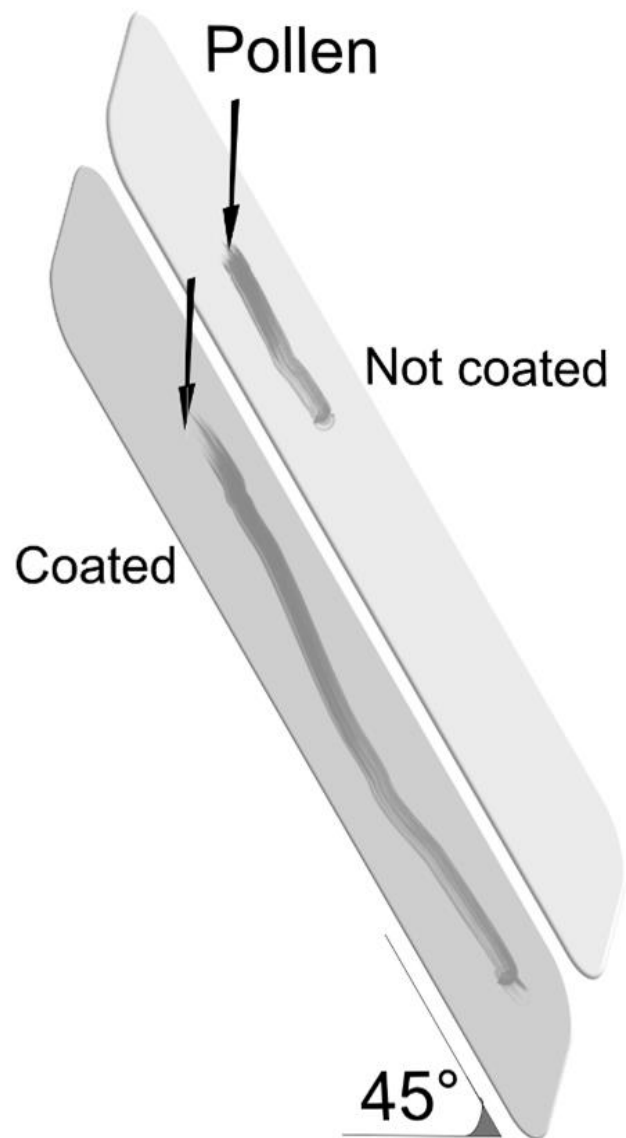**B**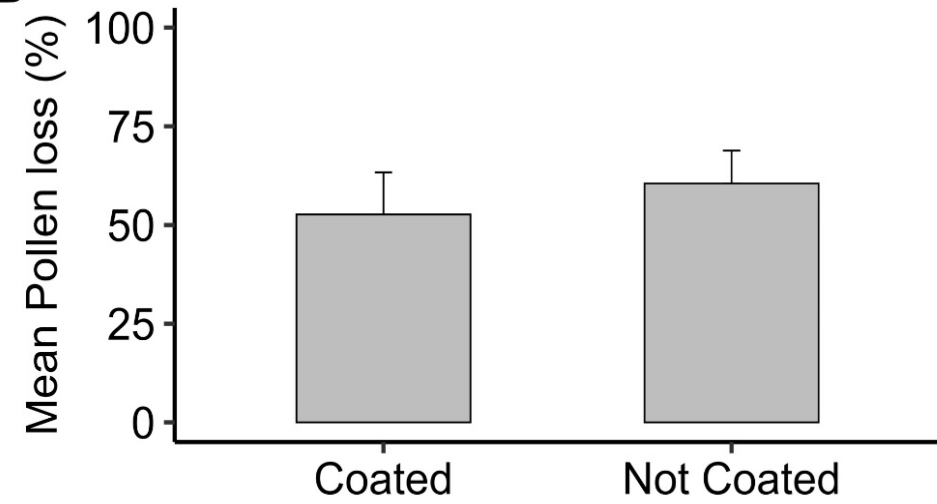**C**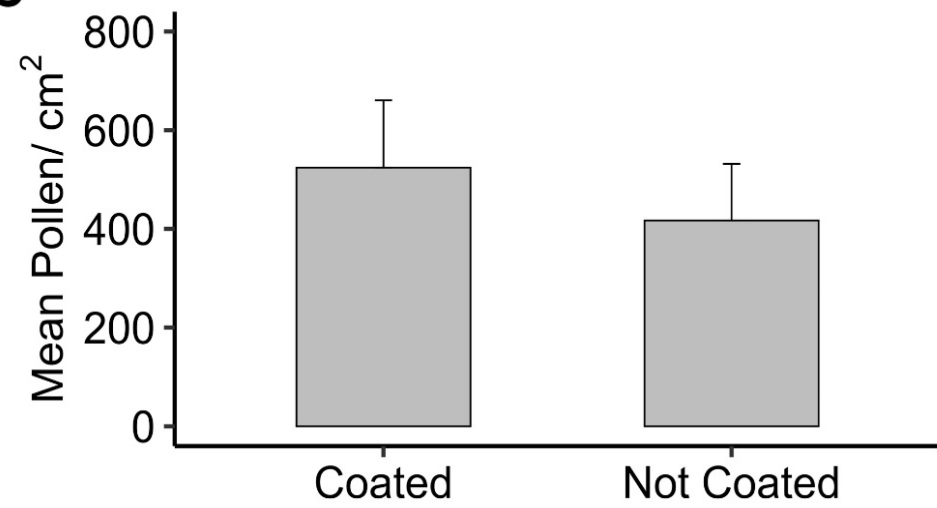

Supplement: S1 Fig — (A) test system “(“slide-friction-test”) to pre-test the effect of a surface change on pollen stickiness (B) effect of a PTFE coating on pollen loss to the surfaces of the Pollen Sedimentation Chamber and (C) effect of coating in pollen deposition on the bottom of the Pollen Sedimentation Chamber. N≥ 3 in all cases. (PDF) [file pone.0259914.s001.pdf]

**A**

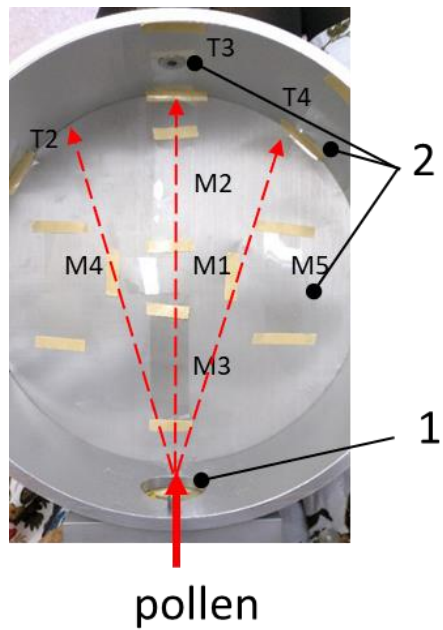

**B**

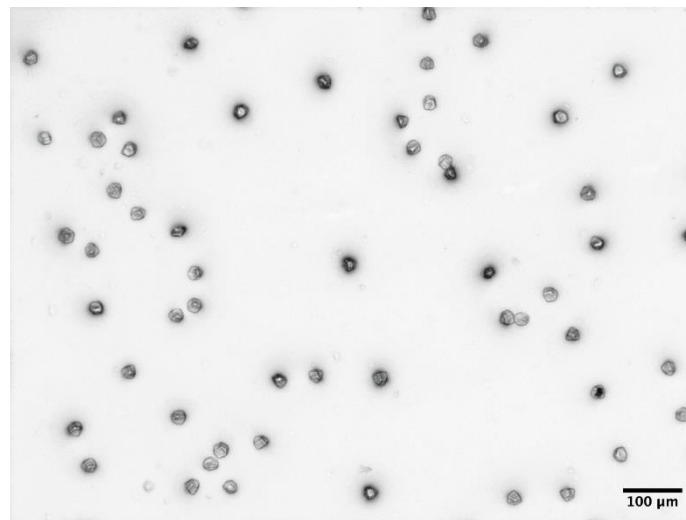

**1 bar**

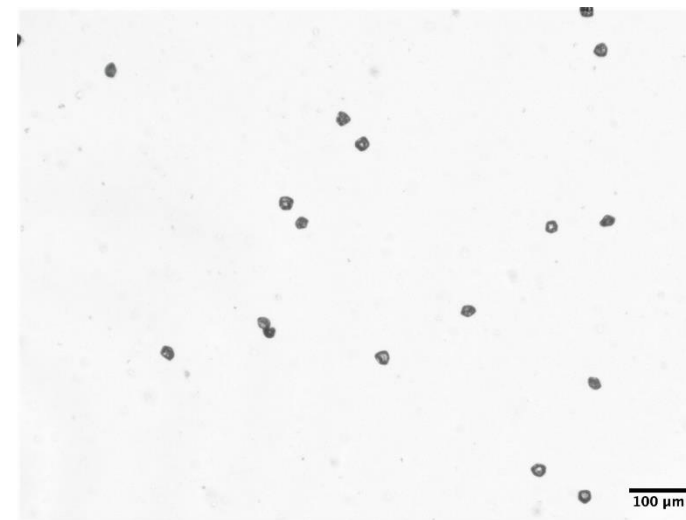

**0.5 bar**

Supplement: S2 Fig — (A) detail of dispersion chamber with route of injected pollen when loading through entrance (1) and examples of Melinex(R) glue tapes (2), (B) sedimented pollen in the position T3, on the dispersion chamber, depending on air pressure. A higher pressure resulted in pollen being propelled against the walls and mesh, where they stuck and were lost. (PDF) [file pone.0259914.s002.pdf]

### Top-level GO Terms, up regulated

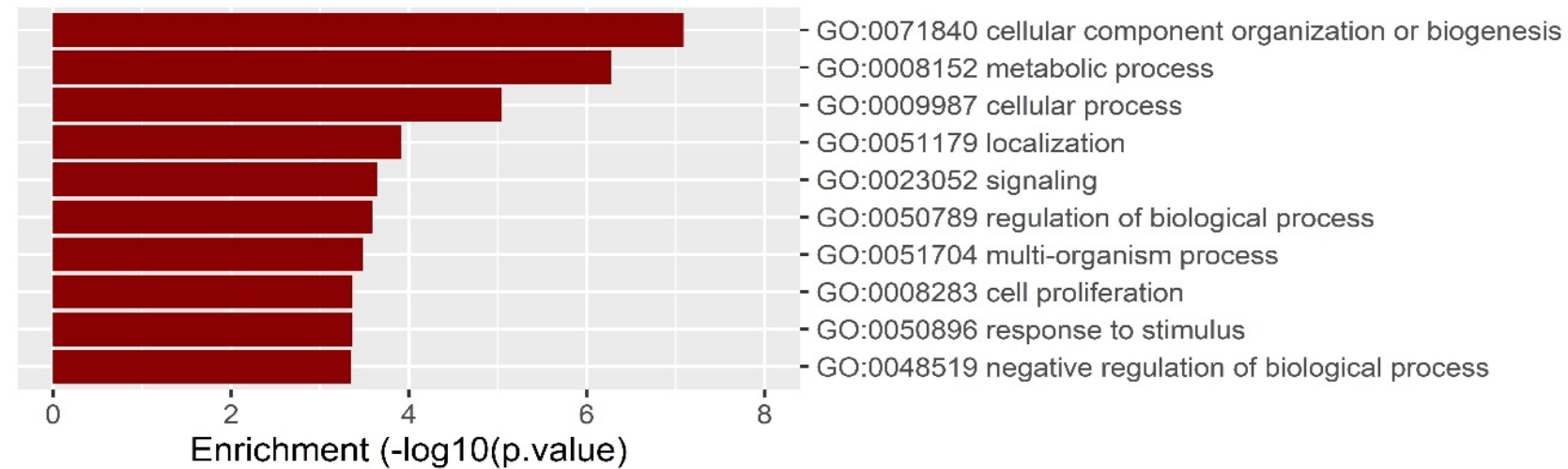

### Top-level GO Terms, down regulated

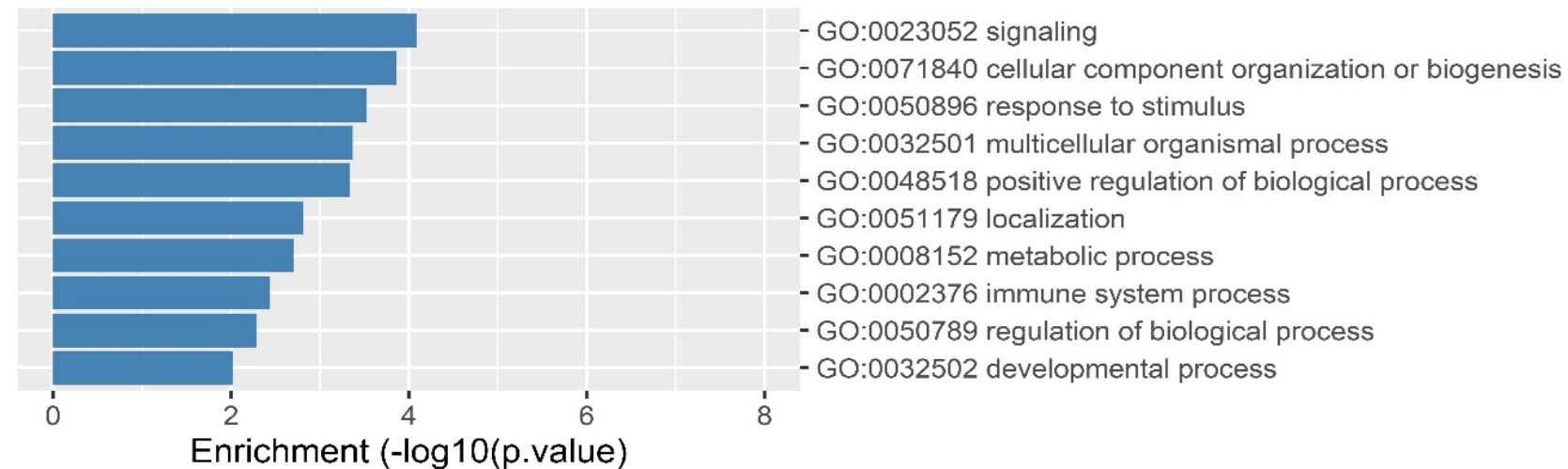

Supplement: S3 Fig — Metascape analysis of the significant regulated genes was performed. GO Terms related to Biological Processes are illustrated. (PDF) [file pone.0259914.s003.pdf]
