# Supplementary material for: Dosing intact birch pollen grains at the air-liquid interface (ALI) to the immortalized human bronchial epithelial cell line BEAS-2B
Source: PLoS One. 2021 Nov 16;16(11):e0259914. doi: 10.1371/journal.pone.0259914 (PMC8594808; doi:10.1371/journal.pone.0259914)
Supplement: S1 Appendix — (DOCX) [file pone.0259914.s004.docx]

**S1 Table 1. Data used to build Fig. 1B**

| Loading tube | Pollen Loss (%) | Mean (%) | SD |
| --- | --- | --- | --- |
| Falcon tube | 65.82 | 69.071 | 19.658 |
|  | 88.78 |  |  |
|  | 78.52 |  |  |
|  | 43.17 |  |  |
| Glass tube | 46.42 | 47.393 | 2.124 |
|  | 48.19 |  |  |
|  | 45.04 |  |  |
|  | 49.92 |  |  |
| No-escape tube | 0.00 | 1.867 | 3.431 |
|  | 0.00 |  |  |
|  | 7.00 |  |  |
|  | 0.47 |  |  |

**S2 Table 2. Data used to build Fig. 1C**

| Loading tube and Air pressure used | Pollen deposited (pollen/cm^2^) | Mean (pollen/cm^2^) | SD |
| --- | --- | --- | --- |
| Falcon tube  1 bar | 326.39 | 368.834 | 162.389 |
|  | 264.24 |  |  |
|  | 609.03 |  |  |
|  | 275.69 |  |  |
| No-escape tube  1 bar | 338.54 | 363.194 | 84.025 |
|  | 256.94 |  |  |
|  | 447.57 |  |  |
|  | 409.72 |  |  |
| No-escape tube  0.5 bar | 581.25 | 579.423 | 32.463 |
|  | 554.17 |  |  |
|  | 624.65 |  |  |
|  | 557.64 |  |  |

**S2 Table 3. Data used to build Fig. 3A**

| Pollen dose | Position on the plate | Mean deposited pollen (pollen/cm^2^) | SD |
| --- | --- | --- | --- |
| 1 mg | Position 1 | 358.333 | 88.462 |
|  | Position 2 | 293.750 | 58.072 |
|  | Position 3 | 339.583 | 23.478 |
|  | Position 4 | 302.778 | 29.191 |
|  | Position 5 | 344.444 | 54.180 |
|  | Position 6 | 320.139 | 6.365 |
| 2 mg | Position 1 | 589.583 | 80.066 |
|  | Position 2 | 625.694 | 87.558 |
|  | Position 3 | 675.694 | 59.561 |
|  | Position 4 | 526.289 | 69.389 |
|  | Position 5 | 477.083 | 52.581 |
|  | Position 6 | 625.694 | 63.749 |
| 4 mg | Position 1 | 1442.361 | 142.131 |
|  | Position 2 | 1134.028 | 106.114 |
|  | Position 3 | 1082.622 | 41.967 |
|  | Position 4 | 1112.500 | 91.073 |
|  | Position 5 | 1243.056 | 37.807 |
|  | Position 6 | 1173.611 | 102.536 |
| 10 mg | Position 1 | 2845.833 | 179.711 |
|  | Position 2 | 2836.806 | 363.888 |
|  | Position 3 | 2884.028 | 150.294 |
|  | Position 4 | 2643.750 | 243.518 |
|  | Position 5 | 2919.444 | 54.500 |
|  | Position 6 | 2876.389 | 31.273 |

**S2 Table 4. Data used to build Fig. 3B**

| Pollen dose | Pollen deposited (pollen/cm^2^) | Mean (pollen/cm^2^) | SD |
| --- | --- | --- | --- |
| 1 mg | 327.43 | 326.505 | 8.718 |
|  | 317.36 |  |  |
|  | 334.72 |  |  |
| 2 mg | 581.25 | 586.690 | 35.557 |
|  | 554.17 |  |  |
|  | 624.65 |  |  |
| 4 mg | 1252.08 | 1205.486 | 40.580 |
|  | 1177.92 |  |  |
|  | 1186.46 |  |  |
| 10mg | 2932.99 | 2834.375 | 145.987 |
|  | 2666.67 |  |  |
|  | 2903.47 |  |  |

**S2 Table 5. Data used to build Fig. 3C**

| Pollen dose | Pollen Loss (%) | Mean (%) | SD |
| --- | --- | --- | --- |
| 1 mg | 39,27 | 39.442 | 0.170 |
|  | 39,44 |  |  |
|  | 39,61 |  |  |
| 2 mg | 45,59 | 46.031 | 2.626 |
|  | 48,85 |  |  |
|  | 43,65 |  |  |
| 4 mg | 37,57 | 40.377 | 2.515 |
|  | 42,42 |  |  |
|  | 41,14 |  |  |
| 10mg | 42,99 | 44.609 | 2.596 |
|  | 47,60 |  |  |
|  | 43,23 |  |  |

**S2 Table 6. Data used to build Fig. 4A**

| Incubation time | Condition | Mean of Cytotoxicity (%) (normalized to control) | SD |
| --- | --- | --- | --- |
| 10 min | Control TW | 1.401 | 3.606 |
|  | Exposed TW | 2.893 | 0.805 |
| 55 min | Control TW | 0.547 | 0.870 |
|  | Exposed TW | 2.923 | 2.079 |
| 2h25 min | Control TW | 1.514 | 1.253 |
|  | Exposed TW | 3.353 | 1.299 |
| 6h50 min | Control TW | 0.034 | 0.683 |
|  | Exposed TW | 2.145 | 0.665 |
| 24 h | Control TW | 2.315 | 6.381 |
|  | Exposed TW | 7.221 | 18.809 |

**S2 Table 7. Data used to build Fig. 4B**

| Incubation time | Condition | Mean of Viability (%) (normalized to control) | SD |
| --- | --- | --- | --- |
| 10 min | Control TW | 97.075 | 5.795 |
|  | Exposed TW | 102.705 | 0.537 |
|  | Positive control | 5.648 | 0.561 |
| 55 min | Control TW | 99.017 | 2.162 |
|  | Exposed TW | 100.537 | 2.638 |
|  | Positive control | 5.466 | 1.183 |
| 2h25 min | Control TW | 103.067 | 2.246 |
|  | Exposed TW | 103.945 | 4.058 |
|  | Positive control | 6.243 | 1.088 |
| 6h50 min | Control TW | 102.178 | 2.947 |
|  | Exposed TW | 103.729 | 1.236 |
|  | Positive control | 6.796 | 0.322 |
| 24 h | Control TW | 99.579 | 4.758 |
|  | Exposed TW | 103.366 | 5.374 |
|  | Positive control | 7.273 | 0.385 |

**S2 Table 8. Data used to build Fig. 4B**

| Incubation time | Gene | Fold change (relative to control) | SD |
| --- | --- | --- | --- |
| 55 min | CXCL2 | 2.50 | 0.58 |
|  | IL6 | 2.91 | 0.73 |
|  | TNFAIP3 | 3.30 | 0.50 |
|  | IL1B | 1.81 | 0.68 |
| 2h25 min | IL1B | 2.32 | 0.55 |
|  | SOCS3 | 2.01 | 0.40 |
| 6h50 min | FOXP1 | 1.26 | 0.13 |
|  | ICAM1 | 2.92 | 0.70 |
|  | IL24 | 4.05 | 0.59 |
|  | IL4R | 1.23 | 0.17 |

**S2 Table 9. Data used to build S1. Fig. (B)**

| **Loading tube** | **Pollen Loss (%)** | **Mean (%)** | **SD** |
| --- | --- | --- | --- |
| Coated | 43,21 | 52.68 | 10.65 |
|  | 73,94 |  |  |
|  | 40,89 |  |  |
| Not Coated | 67,42 | 60.49 | 8.36 |
|  | 70,21 |  |  |
|  | 43,85 |  |  |

**S2 Table 10. Data used to build S1. Fig. (C)**

| **Loading tube** | **Pollen deposited (pollen/cm^2^)** | **Mean (pollen/cm^2^)** | **SD** |
| --- | --- | --- | --- |
| Coated | 640.97 | 524.07 | 136.44 |
|  | 252.08 |  |  |
|  | 679.17 |  |  |
| Not Coated | 326.74 | 417.01 | 114.89 |
|  | 279.17 |  |  |
|  | 645.14 |  |  |
